# Serum Lipidome and Metabolome Alterations in Obese Patients Undergoing Targeted Diet and Exercise Interventions: A Marker of Thyroid Function Recovery?

**DOI:** 10.1155/ije/6065721

**Published:** 2025-09-18

**Authors:** Changxu Zhou, Heng Wang, Qingtao Yu, Jingxin Xin, Huiqi Chen, Ran Zhang, Qingbo Guan, Shanshan Shao

**Affiliations:** ^1^Key Laboratory of Endocrine Glucose & Lipids Metabolism and Brain Aging, Department of Endocrinology, Shandong Provincial Hospital Affiliated to Shandong First Medical University, Ministry of Education, Jinan 250021, Shandong, China; ^2^Shandong Key Laboratory of Endocrine Metabolism and Aging, Jinan 250021, Shandong, China; ^3^Shandong Institute of Endocrine and Metabolic Diseases, Jinan 250021, Shandong, China; ^4^Department of Endocrinology, The People's Hospital of Huaiyin, Jinan 250021, China; ^5^Department of Endocrinology, Shandong Provincial Hospital, Shandong University, Jinan 250021, Shandong, China

**Keywords:** health intervention, metabolism, multiomics, obesity, thyroid hormone

## Abstract

**Purpose:** Dietary and exercise interventions have the potential to modify thyroid hormone levels in individuals with obesity. This study investigates the specific mechanisms through which these interventions influence thyroid function, employing a multiomics approach.

**Methods:** 16 volunteers with obesity participated in a two-week regimen of aerobic exercise combined with dietary control. Fasting blood samples and anthropometric measurements were taken before and after the intervention. Serum untargeted lipidomics and metabolomics analyses were performed, alongside evaluations of serum thyroid hormone levels. Additionally, an RNA sequencing dataset was obtained which included gene expression data for skeletal muscle and subcutaneous fat both prior to and following weight loss.

**Results:** Following the intervention, significant alterations were observed in serum levels of thyroid hormones, lipid molecules, and metabolites among participants. Notably, there was a substantial reduction in the levels of tyrosine and phenylalanine (*p* < 0.001). Furthermore, this intervention had a pronounced effect on the activity of thyroid hormone signaling pathways.

**Conclusion:** Dietary modifications along with exercise facilitate the restoration of thyroid function by enhancing the consumption of tyrosine and phenylalanine while concurrently altering the activity within thyroid hormone signaling pathways. These findings provide valuable insights into potential treatments for obesity-related thyroid dysfunction.

**Trial Registration:** Chinese Registry of Clinical Trials: ChiCTR2000040981

## 1. Introduction

Obesity is a chronic metabolic disorder characterized by an abnormal increase in the proportion of body fat relative to total body mass and/or excessive accumulation of localized adipose tissue [1]. It serves as a significant risk factor for the onset and progression of numerous diseases, including type 2 diabetes, hyperlipidemia, renal disorders, nonalcoholic fatty liver disease, and cardiovascular ailments [2–4]. The World Health Organization has identified obesity as a global chronic disease threatening human health. Recent statistics indicate that over 2 billion individuals worldwide are either overweight or obese, constituting approximately 30% of the global population [5, 6]. The situation regarding weight control is progressively worsening.

In recent years, the association between obesity and thyroid dysfunction has been widely concerned. Research has demonstrated that the risk of subclinical hypothyroidism is enhanced by 70% among obese individuals, and the risk of clinical hypothyroidism is augmented by more than twice [7], which could be attributed to the ectopic accumulation of excessive lipids in thyroid follicular epithelial cells [8, 9]. Animal experiments have likewise affirmed that obesity leads to ectopic deposition of thyroid lipids, alters the morphology and structure of the thyroid, and subsequently influences the levels of serum thyroid hormones [10]. Furthermore, obesity is acknowledged as a risk factor for thyroid autoimmunity. The incidence of autoimmune thyroid disease in obese populations is conspicuously high, with reports suggesting that it affects 12.4% of children and ranges from 10% to 60% among adults [11]. Obesity has been recognized as a factor contributing to both peripheral and central thyroid hormone resistance [12]. Weight loss strategies, such as bariatric surgery and low-calorie dietary interventions, have manifested efficacy in reversing hyperthyrotropinemia in obese individuals [13].

Aerobic exercise and dietary intervention have been attested to be effective in reversing obesity and its associated complications. However, there are still significant gaps in the current literature regarding the impact of such intervention on thyroid function. Current research has noted a decrease in free triiodothyronine (FT3) levels among subjects after weight loss; nevertheless, the changes in free thyroxine (FT4) and thyroid-stimulating hormone (TSH) levels have been inconsistent. The underlying mechanisms governing these thyroid hormone alterations remain elusive [14, 15]. Moreover, no study has integrated untargeted lipidomics and metabolomics with in silico mining of public transcriptomic atlases to elucidate the molecular pathways linking weight loss to thyroid function recovery in obese humans. Furthermore, the potential for identifying predictive biomarkers for early risk of hypothyroidism within a multiomics framework remains unexplored.

To elucidate the changes in thyroid function following weight loss, we employed untargeted lipidomics and metabolomics techniques to characterize the alterations in plasma lipids and metabolites among obese individuals after an intervention involving low-to-moderate intensity aerobic exercise combined with dietary control, and a comparable public dataset was used to further explore the changes in thyroid function under intervention. We aimed to (i) uncover novel lipid and metabolite trajectories associated with improved thyroid function, (ii) delineate key metabolic pathways that may mechanistically explain these improvements, and (iii) generate candidate biomarkers for early identification of individuals at high risk of hypothyroidism. We anticipate that this investigation will not only provide new insights into the metabolic states and pathway dynamics associated with obesity but also facilitate the identification of at-risk populations for hypothyroidism, thereby contributing to the development of innovative preventive strategies against this condition.

## 2. Materials and Methods

### 2.1. Research Design

All participants were obese individuals who participated in the Weight Management Summer Camp, an initiative organized by the Department of Endocrinology at Shandong Provincial Hospital Affiliated to Shandong First Medical University, during July 2017 and July 2018. The inclusion criteria consisted of a body mass index (BMI) of 30 kg/m^2^ or higher, or a waist circumference measuring 85 cm or more for women and 90 cm or more for men. The exclusion criteria included (a) a preexisting diagnosis of hypertension or coronary heart disease; (b) a confirmed or suspected history of tumors; (c) use of medications that affect thyroid function or blood lipid levels (such as statins, thyroid hormone analogs, antithyroid drugs, amiodarone, lithium, epinephrine, and beta-blockers) within the preceding 3 months; (d) pregnancy, severe liver dysfunction (alanine aminotransferase [ALT] or aspartate aminotransferase [AST] > 100 IU/L), or abnormal renal function (creatinine > 105 μmol/L; glomerular filtration rate < 60 mL/min); and (e) incomplete data regarding age, sex, BMI, or thyroid function.

Participants engaged in a two-week intervention that incorporated moderate to low-intensity aerobic exercise alongside dietary control. Exercise was performed twice daily (morning and afternoon, 7 days per week) and comprised walking, jogging, aerobics, table tennis, and badminton, each lasting 2 h. The intensity of the exercises was predominantly moderate to low, with postexercise heart rates ranging from 100 to 120 beats per minute. All physical activities were conducted under the supervision of trained personnel. The dietary intervention consisted of an energy-restricted plan providing either 1400 or 1600 kcal per day based on each individual's basal metabolic rate. The macronutrient distribution was designed to consist of 65% carbohydrates, 20% lipids, and 15% proteins. This diet emphasized increased consumption of vegetables, fish, and other white meats while limiting oils, salt intake, and red meat. Anthropometry (height, weight, waist circumference, and blood pressure) and visceral fat content were recorded between 8 a.m. and 10 a.m., following a fasting period of 10 h both prior to and subsequent to the intervention. Fasting blood samples were also collected for analysis.

All adult participants as well as juvenile participants along with their guardians received comprehensive information regarding the study's objectives and procedures; they provided written informed consent before data collection commenced. The design and methodology employed in this study adhere strictly to the principles outlined in the Declaration of Helsinki (1975) and have been approved by the Ethics Committee at Shandong Provincial Hospital affiliated with Shandong First Medical University (LCYJ: NO. 2017-029).

### 2.2. Anthropometric Measurements

All measurements were performed in the fasting state by trained staff at Shandong Provincial Hospital Affiliated to Shandong First Medical University. Height and weight were measured using a height and weight meter, while BMI was calculated by dividing the weight in kilograms by the square of the height in meters (BMI = kg/m^2^). Waist circumference was assessed with a flexible yet inelastic ruler positioned 1 cm above the navel. After a 15-minute seated rest, blood pressure was measured three times using an electronic sphygmomanometer, ensuring at least one minute between each reading. The average of these three measurements was subsequently recorded. The content of visceral fat was quantified utilizing a human body composition analyzer (InBody 770 device, Korea).

### 2.3. Blood Collection and Laboratory Testing

The collection of blood samples and the subsequent laboratory procedures were meticulously carried out by trained personnel. Blood was drawn from the median cubital vein, followed by the necessary separation of serum and plasma, and stored at −80°C until analysis. Serum levels of FT3, FT4, and TSH were quantitatively assessed using electrochemiluminescence immunoassay (Cobas E601, Roche, Basel, Switzerland). An automatic biochemical analyzer (BECKMAN Chemistry Analyzer AU5800 System, Beckman Coulter, Tokyo, Japan) was utilized to measure AST, ALT, fasting plasma glucose (FPG), triglycerides (TGs), total cholesterol (TC), and low-density lipoprotein cholesterol (LDL-C). This comprehensive approach ensured the accuracy and reliability of the biochemical evaluations conducted as part of this study.

### 2.4. Lipidomics and Metabolomics Tests

A total of 32 samples from 16 volunteers were submitted for untargeted lipidomics and metabolomic analysis conducted by LipidALL Technologies Co. Ltd. The lipid components of the samples were analyzed using electrospray ionization in conjunction with normal-phase high-performance liquid chromatography (HPLC). An enhanced Bligh/Dyer extraction method was employed to optimize lipid extraction, supplemented with appropriate internal standards (PC-d31, PE-d31, PS-d31, PI-d31, PA-d31 [16], and a series of specific isotopically labeled standards such as d5-TAG, d5-DAG, and cholesteryl-2,2,3,4,4,6-D6 Octadeconate). These standards played a crucial role in the quantification of various lipid species [17]. The analysis of polar lipids was performed utilizing mass spectrometry with multireaction monitoring transitions for comparative assessment [18]. Following treatment with a specific HPLC buffer for serum samples, metabolic data were analyzed in electrospray ionization mode using an Agilent 1290 Infinity II UPLC coupled with a QTOF 5600 PLUS mass spectrometer. This advanced analytical setup enabled comprehensive profiling and quantification of lipid species and contributed to an enhanced understanding of the metabolic characteristics exhibited by the study participants.

### 2.5. Lipidomics and Metabolomics Data Analysis

Principal component analysis (PCA): PCA is a technique that enhances the information content of the original dataset by identifying a set of new, uncorrelated variables. This transformation facilitates improved visualization and comprehension of the data.

Orthogonal partial least-squares discrimination analysis (OPLS-DA): OPLS-DA effectively eliminates data variation that is not related to categorical variables within the independent variables. This process allows classification information to be predominantly concentrated in a single principal component, thereby simplifying the model and enhancing its interpretability. Consequently, the discriminative power of the model becomes more pronounced.

Metabolite set enrichment analysis (MSEA): MSEA is employed to evaluate the extent of enrichment of specific metabolite groups within biological samples [19]. In this study, we identified differentially expressed metabolites and conducted MSEA analysis to elucidate the metabolic pathways that play pivotal roles in biological regulation.

Metabolic pathway analysis (MetPA): MetPA is an integrated approach that combines overrepresentation analysis with pathway topological analysis, facilitating the identification of the most pertinent metabolic pathways involved in a specific metabolomics study [20]. In this investigation, we focus on metabolites that exhibit significant differences and correspond to matching Kyoto Encyclopedia of Genes and Genomes (KEGG) IDs.

### 2.6. Transcriptome Data Analysis

#### 2.6.1. Data Selection and Processing

The gene expression profile of GSE205891 [21], a dataset derived from RNA sequencing and closely related to our study, was retrieved from the Gene Expression Omnibus database maintained by the National Center for Biotechnology Information. This dataset comprises 62 samples of skeletal muscle and subcutaneous fat tissue obtained from individuals with obesity and type 2 diabetes. These samples were collected both prior to and following the participants' engagement in a diet and exercise intervention, alongside standard care. To process the downloaded data, we utilized the “limma” package from the Bioconductor project, which facilitated the normalization of RNA sequencing data [22]. We calculated fold changes between pre- and postintervention samples and derived corresponding *p* values to quantify differential expression. To enhance our identification of differentially expressed genes (DEGs) in skeletal muscle and subcutaneous fat, we employed robust rank aggregation (RRA) analysis. This method is robust for integrating and meta-analyzing gene lists, providing a comprehensive assessment of gene expression changes in response to the intervention [23]. By applying RRA, we aimed to consolidate and validate findings from individual analyses, thereby strengthening the reliability of our conclusions regarding the molecular effects of diet and exercise on thyroid function recovery.

#### 2.6.2. Enrichment Analysis

To elucidate the functional implications of the DEGs, we utilized the R software package clusterProfiler Version 3.16.1 for pathway enrichment analysis based on KEGG pathways. ClusterProfiler is a versatile enrichment tool specifically designed for functional and comparative studies, allowing us to gain insights into the biological processes and pathways influenced by these DEGs [24].

#### 2.6.3. Pathway Deregulation Score (PDS)

To assess the alterations in specific biological pathways before and after the intervention, we employed the “Pathifier” package from the Bioconductor project to calculate the PDS. The PDS quantifies the degree of pathway dysregulation, with values ranging from 0 to 1; higher scores indicate a greater extent of pathway perturbation relative to the control group [25]. Utilizing Student's *t*-test, we compared the PDS distributions between pre- and postintervention groups to statistically evaluate whether the intervention had a significant impact on these pathways.

### 2.7. Statistical Analyses

Quantitative data were presented as means and standard deviations (mean ± SEM). The Shapiro–Wilk test was employed to assess the normality of the data distribution. To evaluate clinical significance before and after the intervention, either the paired Student's *t*-test or the Wilcoxon signed-rank test was utilized. Given the exploratory nature of the study and the limited sample size, we did not apply formal multiple-testing corrections. Instead, false-positive risk was mitigated by combining fold-change filters with cross-validation against biochemical data. Thus, unless stated otherwise, *p* < 0.05 was considered statistically significant. For lipidomics and metabolomics data, we applied the Wilcoxon signed-rank test, categorizing metabolites based on a significance threshold of *p* < 0.05 and a fold change criterion greater than or less than 1. In our transcriptome analysis, DEGs were identified using a threshold of log2|fold change| > 0.1 and a *p* value of < 0.05.

Statistical computations were performed using SPSS Version 26, while data processing and advanced statistical analyses were conducted with R Version 4.2. Graphical representations of our findings were generated using GraphPad Prism 8.0 software.

## 3. Results

### 3.1. Effects of Intervention on Glucose and Lipid Metabolism and Thyroid Function

**Figure 1:**
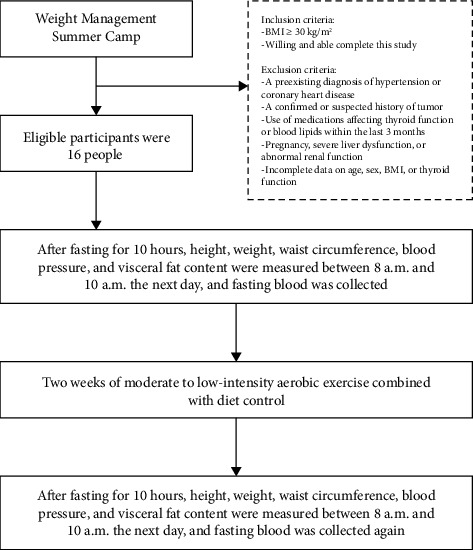
Research process.

Study flow is summarized in Figure 1. We recruited a cohort of 16 volunteers with obesity as research subjects, consisting of 9 males and 7 females, aged between 10 and 45 years, with a mean age of 21.15 ± 9.01 years.  Table 1 presents the anthropometric measurements and serological parameters of these subjects both prior to and following a regimen of moderate to low-intensity aerobic exercise combined with dietary control.

Significant reductions (*p* < 0.05) were observed in waist circumference, body weight, BMI, and visceral fat content among the subjects after a two-week intervention period. These results unequivocally demonstrate the efficacy of the combined intervention in promoting weight loss and enhancing body composition. The changes noted underscore the potential of this approach as an effective strategy for managing obesity and associated metabolic health issues. Furthermore, our analysis indicated that the intervention resulted in significant reductions (*p* < 0.05) in blood glucose levels, TGs, and TC postintervention, thus corroborating the beneficial impact of moderate to low-intensity exercise alongside dietary control on improving glucose and lipid metabolism in obese individuals. Notably, alongside weight loss outcomes, we also observed alterations in markers indicative of thyroid function. Specifically, there was a significant increase in FT4 levels, rising from 16.21 ± 2.07 pmol/L prior to the intervention to 17.58 ± 2.07 pmol/L postintervention (*p*=0.005). In contrast, FT3 levels showed a substantial decrease from 5.59 ± 1.69 pmol/L preintervention to 4.20 ± 0.74 pmol/L postintervention (*p*=0.004). Additionally, the FT3/FT4 ratio exhibited a notable decline from 0.34 ± 0.08 before the intervention to 0.24 ± 0.05 after the intervention (*p* < 0.001). While TSH levels displayed a downward trend, this change did not achieve statistical significance (*p*=0.187). These findings suggest that the intervention not only influences metabolic parameters but also affects thyroid hormone dynamics, potentially impacting overall metabolic regulation in obese subjects.

### 3.2. Effect of Intervention on Serum Lipid Species

Serum biochemical assessments initially indicated that the intervention led to a significant reduction in serum cholesterol and TG levels among the volunteers. Acknowledging the complex and multifaceted nature of lipid metabolism, we performed comprehensive serum lipidomics analyses to explore the nuanced changes in various lipid species, including fatty acyls, glycerophospholipids, sphingolipids, and neutral lipids. PCA of the lipidomic data revealed a clear trend of separation in lipid profiles between pre- and postintervention states. This finding suggests that the combination of exercise and dietary control induced a substantial transformation in the lipidomic landscape of the subjects (Supporting Figure 1).

**Figure 2:**
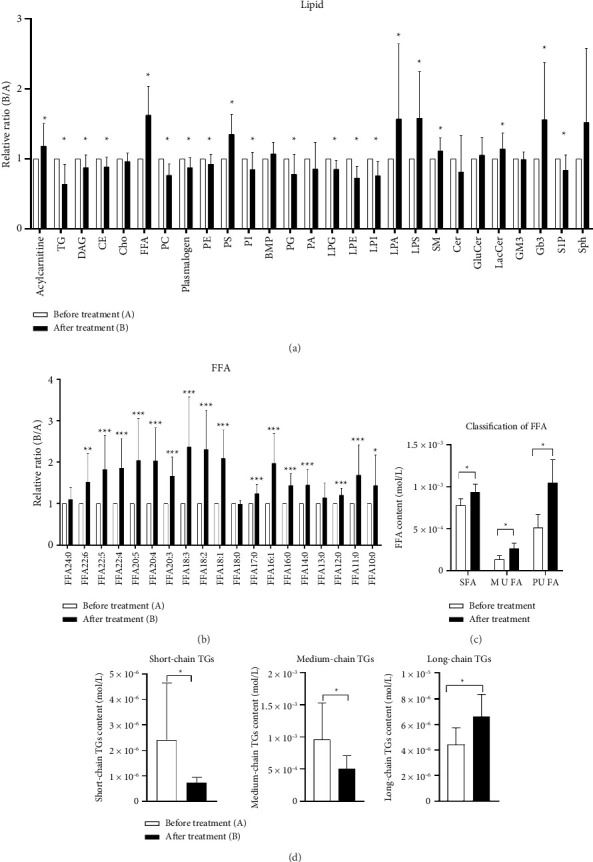
Lipid changes after intervention. (a) Lipid molecules change before and after intervention. TG: triacylglycerol, DAG: diacylglycerol, CE: cholesteryl ester, Cho: free cholesterol, FFA: free fatty acid, PC: phosphatidylcholine, PE: phosphatidylethanolamine, PS: phosphatidylserine, PI: phosphatidylinositol, BMP: bis (monoacylglycerol) phosphate, PG: phosphatidylglycerol, PA: phosphatidic acid, LPC: lysophosphatidylcholine, LPE: lysophosphatidylethanolamine, LPI: lysophosphatidylinositol, LPA: lysophosphatidic acid, LPS: lysophosphatidylserine, SM: sphingomyelin, Cer: ceramide, GluCer: glucosylceramide, LacCer: lactosylceramide, GM3: monosialodihexosylganglioside, GB3: trihexosylceramide, S1P: sphingosine-1-phosphate, and Sph: sphingosine. (b) Changes of serum FFAs before and after intervention. FFA: free fatty acid. (c) Changes of SFAs, MUFAs, and PUFAs before and after the intervention. FFA: free fatty acid, SFA: saturated fatty acid, MUFA: monounsaturated fatty acid, and PUFA: polyunsaturated fatty acid. (d) TG changes with different chain lengths. TGs: triglycerides. ^∗^*p* < 0.05, ^∗∗^*p* < 0.01, and ^∗∗∗^*p* < 0.001.

A total of 669 lipid molecules were identified in a serum untargeted lipidomics analysis encompassing 27 lipid families. Among these, 420 lipid molecules and 20 lipid families exhibited significant changes following the intervention. As illustrated in Figure 2(a), the lipids that showed marked increases included acylcarnitine, free fatty acids (FFAs), phosphatidylserine (PS), trihexosylceramide, lysophosphatidic acid (LPA), lysophosphatidylserine (LPS), sphingomyelin (SM), and lactosylceramide. Conversely, the lipids that decreased significantly comprised TGs, glycerol diesters, cholesteryl esters, phosphatidylcholine (PC), phosphatidylethanolamine (PE), phosphatidylinositol (PI), phosphatidylglycerol (PG), plasmalogens, lysophosphatidylethanolamine (LPE), lysophosphatidylcholine (LPC), lysophosphatidylinositol (LPI), and sphingosine-1-phosphate.

#### 3.2.1. FFA

After the intervention, there was an overall increasing trend in serum FFAs. A total of 19 FFAs were identified, comprising 9 saturated fatty acids (SFAs), 2 monounsaturated fatty acids (MUFAs), and 8 polyunsaturated fatty acids (PUFAs) (Figure 2(b)). Postintervention, the levels of 16 types of FFAs were significantly elevated compared to preintervention values (*p* < 0.05), with FFA18:1 and FFA20:3 exhibiting the most pronounced increases (*p* < 0.001). As illustrated in Figure 2(c), the total concentrations of MUFAs, SFAs, and PUFAs all showed significant increases relative to their preintervention levels (*p* < 0.05).

#### 3.2.2. TG

A comprehensive analysis of a panel comprising 150 TG molecules was conducted, revealing a significant overall decline in total TG levels following the intervention (*p* < 0.05). To elucidate the specific alterations in TG composition, we categorized the TGs based on their carbon chain length into short-chain TGs (44–46 total carbon atoms), medium-chain TGs (48–56 total carbon atoms), and long-chain TGs (≥ 58 total carbon atoms) [26]. Consistent with the general reduction in total TG levels postintervention, both short-chain and medium-chain TGs demonstrated significant decreases compared to their preintervention values. In contrast, long-chain TGs exhibited a notable increase relative to their preintervention state (Figure 2(d)).

#### 3.2.3. Phospholipids and Hemolytic Phospholipids

After the intervention, the serum levels of PC, PE, PI, and PG were significantly reduced (*p* < 0.05). Conversely, a subset of phospholipid molecules demonstrated a notable increase following the intervention, with concentrations exceeding 1.5 times their preintervention levels (Supporting Figure 2). In terms of lysophospholipids, LPA and LPS exhibited significant increases postintervention, while those of LPI, LPE, and LPC showed decreases (*p* < 0.05). Notable variations among different species of lysophospholipids were also recorded. All LPS molecules increased in concentration, with LPS 18:0 showing the most pronounced elevation; conversely, both LPI and LPE experienced reductions, particularly for LPI 20:3 and LPE 18:2 which displayed the most significant declines (Supporting Figure 3).

#### 3.2.4. Sphingolipid

Sphingolipids, primarily consisting of SM and ceramide (Cer), exhibited distinct responses following the intervention. A thorough analysis of 31 SM and 24 Cer species revealed that 19 SM and 7 Cer molecules experienced significant changes postintervention (*p* < 0.05). Notably, the dynamics of SM were largely inversely related to those of phospholipids; specifically, there was a substantial increase in SM levels after the intervention, whereas Cer concentrations significantly decreased (*p* < 0.05) (Supporting Figure 4).

### 3.3. Impact of Intervention on the Metabolome

**Figure 3:**
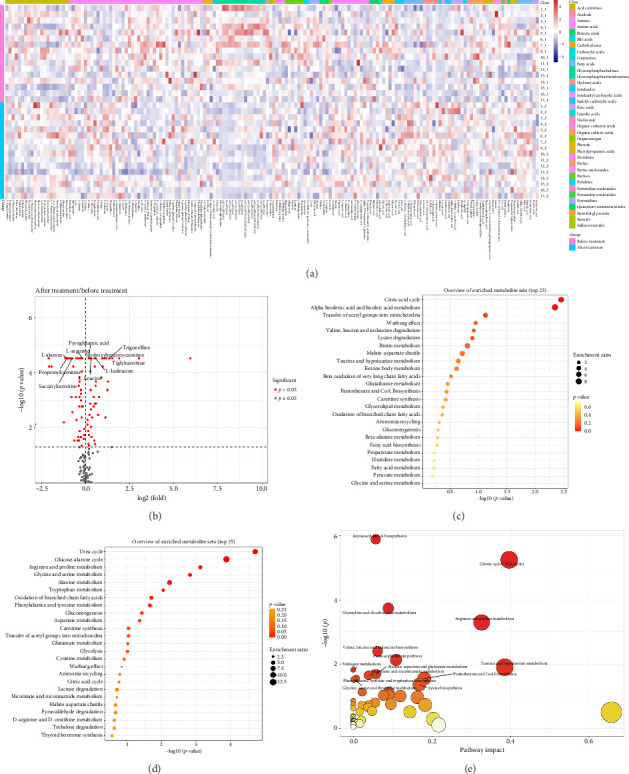
Changes of metabolites after intervention. (a) Heat map of metabolite change. (b) Volcanic map of metabolite change. (c) Dot plot of MSEA analysis of upregulated metabolites. (d) Dot plot of MSEA of downregulated metabolites. (e) MetPA of differential metabolites.

PCA and OPLS-DA score maps revealed significant alterations in the lipid profiles of subjects following the intervention, as illustrated in Supporting Figure 5. More than 90 metabolites exhibited notable changes, which were visually represented through heat maps (Figure 3(a)) and volcano plots (Figure 3(b)). Among the metabolites displaying the most pronounced variations were several amino acids, including isoleucine, pyroglutamic acid, and tiglylcarnitine. Notably, levels of phenylalanine and tyrosine decreased significantly (*p* < 0.05).

To further investigate the interplay between these metabolites and human physiology, we employed two pathway analysis methodologies. The results from MSEA indicated that pathways related to the citric acid cycle as well as alpha-linolenic acid and linoleic acid metabolism were enriched among those with increased metabolite content. Conversely, phenylalanine and tyrosine metabolism pathways were enriched in metabolites exhibiting reduced content. Additionally, although not reaching statistical significance, our analysis suggested a potential involvement of the thyroid hormone synthesis pathway (*p* = 0.262), with tyrosine identified as an enriched metabolite (Figures 3(c) and 3(d)). The MetPA analysis presented in Figure 3(e) indicated that the postintervention metabolite alterations involved several metabolic pathways, including citrate cycle (TCA cycle); arginine biosynthesis; glyoxylate and dicarboxylate metabolism; and valine, leucine, and isoleucine biosynthesis; along with phenylalanine, tyrosine, and tryptophan biosynthesis.

### 3.4. Validation of Intervention on Thyroid Function

**Figure 4:**
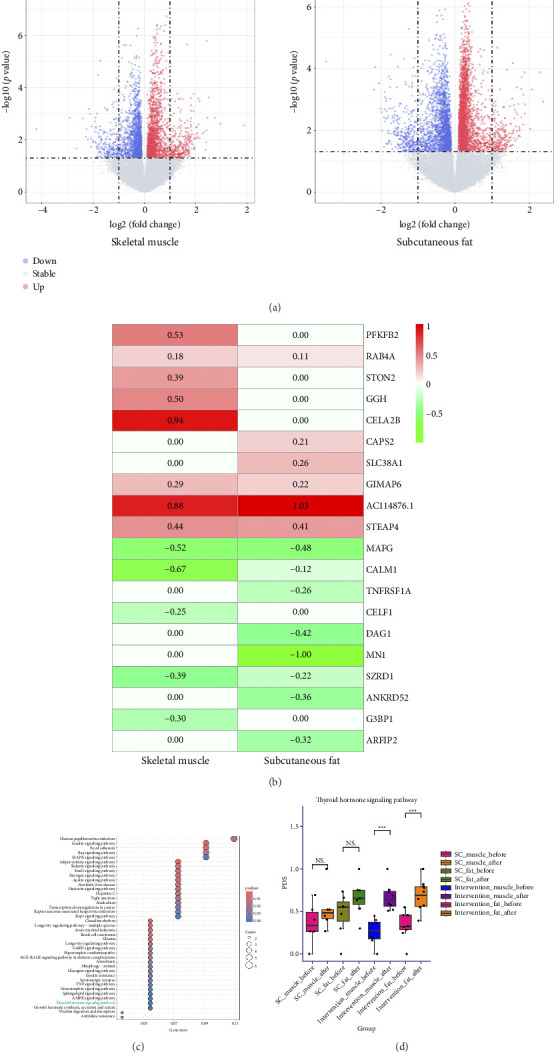
Impact of diet and exercise intervention on thyroid function. (a) Volcano map of DEGs in different tissues of GSE205891. (b) DEGs in subcutaneous fat and skeletal muscle with RRA. (c) KEGG pathway enrichment of DEGs in RRA. (d) PDS of thyroid hormone signaling pathway in different interventions. SC: standard care; PDS: pathway deregulation score. ^∗∗∗^*p* < 0.001.

To elucidate the effects of dietary and exercise interventions on thyroid function, we specifically focused on the transcriptomic analysis of two key thyroid hormone effector organs: skeletal muscle and subcutaneous adipose tissue. We utilized dataset GSE205891, which encompasses data from patients with obesity and diabetes regarding their skeletal muscle and subcutaneous fat, collected after eight months of either a diet and exercise intervention or standard care. Initially, our attention was directed toward samples from the diet and exercise intervention group. By applying thresholds of |logFC| > 0.1 and *p* < 0.05, we identified two clusters of DEGs, which were visually represented using volcano plots (Figure 4(a)). Subsequently, RRA analysis was conducted with a significance threshold set at *p* < 0.05, ultimately revealing 127 significantly DEGs (Figure 4(b)).

To explore the functional implications of these differential genes, we performed KEGG pathway enrichment analysis (Figure 4(c)). The results indicated significant enrichment in the thyroid hormone signaling pathway, identifying *PFKFB2*, *HRAS*, and *MED13* as key contributors. Notably, *HRAS* expression was downregulated in both skeletal muscle and subcutaneous adipose tissue; conversely, *PFKFB2* exhibited upregulation in skeletal muscle while *MED13* showed downregulation within the same tissue type. However, in the standard care group, changes observed in subcutaneous fat for *MED13* were consistent with those noted in the diet and exercise intervention group. Additionally, no significant differences were detected for other genes before versus after 8 months of intervention (Supporting Figures 6–8).

Finally, to gain a deeper understanding of the direct relationship between diet and exercise intervention and thyroid function recovery, we compared the PDS distribution of the thyroid hormone signaling pathway before and after different intervention methods using the Pathifier algorithm. We found that the activity of thyroid hormone signaling pathway in skeletal muscle and subcutaneous fat did not change under standard care before and after the intervention. In contrast, after 8 months of diet-exercise intervention, the activity of thyroid hormone signaling pathway in both tissues exhibited significant differences (Figure 4(d)). The finding that the thyroid hormone signaling pathway and its gene expression vary across different interventions supports our hypothesis that diet and exercise interventions promote thyroid function recovery.

## 4. Discussion

Following a two-week regimen of moderate to low-intensity aerobic exercise combined with dietary intervention, the obese subjects in this study demonstrated significant improvements across multiple indices when compared to their baseline measurements. BMI, waist circumference, visceral fat area, FPG, TGs, and TC all declined—changes that mirrored previous weight loss studies and were known to attenuate metabolic syndrome [27]. Thyroid hormone dynamics also shifted: obese subjects exhibited a decrease in FT3 levels alongside an increase in FT4 following the intervention; TSH levels also showed a downward trend, although this did not reach statistical significance. Research has established that elevated FT4 levels correlate with metabolic benefits, whereas higher TSH levels are linked to increased metabolic risk [28]. We hypothesize that these alterations may reflect an enhancement in thyroid hormone synthesis function and a reduction in thyroid hormone resistance subsequent to weight loss.

Untargeted lipidomics and metabolomic profiling revealed extensive remodeling of serum lipids and metabolites after the combined weight loss intervention. Despite the well-described association between reduced thyroid function and elevated serum TG levels [29], our participants experienced a substantial increase in serum fatty acid concentrations alongside a marked decrease in total TG levels. A particularly noteworthy finding was the rise in long-chain TG content. Previous research has indicated that individuals who engage in regular physical activity tend to exhibit a lower overall trend in TG levels compared to their sedentary counterparts, along with a significant increase in two specific types of long-chain TGs [30]. We hypothesize that this phenomenon may be attributed to distinct metabolic mechanisms governing TGs of varying chain lengths. Medium-chain TGs, whose fatty acids can traverse membranes without transporters or cytosolic binding proteins, were rapidly hydrolyzed by lipases and oxidized in mitochondria to meet immediate energy demands [31, 32]. In contrast, long-chain fatty acids necessitated the assistance of fatty acid–binding proteins for cellular uptake, intracellular transport, regulatory functions, and metabolism [33]. We posit that during the initial phases of weight loss interventions, the body predominantly utilizes short and medium-chain TGs. Meanwhile, long-chain TGs, due to their greater carbon content and more intricate metabolic pathways, may not be as actively engaged in metabolism. Phospholipids were pivotal in cellular activation, maintaining basal metabolic and hormonal balance, and bolstering human immunity and regeneration. Beyond these roles, phospholipids also facilitated fat metabolism, mitigated serum cholesterol levels, enhanced blood circulation, and deterred the onset of cardiovascular diseases and fatty liver. The alterations in lipid species, such as PC and SM, were linked to weight loss outcomes in obese individuals subsequent to dietary management [34]. Despite these insights, the precise biological functions of individual lipid metabolites remain incompletely understood and warrant further investigation.

Metabolic profiling revealed that differential metabolites were significantly enriched in pathways related to sugars, amino acids, and fatty acids. In particular, the coordinated decline in phenylalanine and tyrosine recapitulated previous associations with successful weight loss [35]. Notably, phenylalanine acted as a precursor to tyrosine, which is an essential substrate for the synthesis of thyroid hormones and was often regarded as a potential biomarker for thyroid disorders [36]. Therefore, the observed reduction in phenylalanine and tyrosine levels within the body may indicate an upregulation of thyroid hormone synthesis along with alterations in thyroid function. The decrease in phenylalanine and tyrosine could reflect a metabolic shift toward increased thyroid hormone activity, subsequently influencing energy expenditure and fat metabolism.

Thyroid hormones regulate metabolic processes through various tissues, including skeletal muscle and subcutaneous fat [37]. By examining these tissues in conjunction, a more comprehensive understanding of thyroid function can be attained. Transcriptomic analysis confirmed that thyroid hormone signaling pathway was remodeled in a favorable direction. Expression of the thyroid oncogenes *HRAS* declined, while glycolytic genes, such as *PFKFB2*, were upregulated. *HRAS*, a distinct subtype within the Ras GTPase family implicated in thyroid cancer [38, 39], had demonstrated that its expression in subcutaneous fat diminishes when caloric intake is restricted, thereby mitigating cancer signaling pathways in murine models [40]. *PFKFB2* played a crucial role in glycolysis [41], and its elevated levels in the heart could prevent cardiomyopathy while providing systemic metabolic benefits [42]. Studies had demonstrated that *PFKFB2* expression increases following exercise in high-fructose–fed mice [43]. Additionally, *PFKFB2* activity was significantly reduced in hypothyroid rats but partially recovers with triiodothyronine treatment [44]. *MED13* was a component of the mediator complex that controlled cell cycle regulation and development in a tissue-specific manner and exhibited reciprocal regulation [45]. Overexpression of cardiac *MED13* had been shown to prevent weight gain [46], whereas the loss of skeletal muscle *MED13* led to enhanced glucose uptake and glycogen deposition, protecting the liver from steatosis [47]. *MiR-208a* negatively regulated *MED13* [48]; although the increase did not reach statistically significant, we observed that *miR-208a* levels in skeletal muscle increased following intervention (logFC = 1.12, *p*=0.16). This upregulation might account for the observed decrease in *MED13* expression within skeletal muscle.

Dietary modification is a cornerstone for preventing and managing lipid-related metabolic disorders. Our funding further demonstrates that the combination of dietary modification and exercise not only facilitates weight loss but also may restore thyroid function, thus offering a new avenue for primary prevention and etiological diagnosis in obese individuals with hypothyroidism. Through this intervention, we enhanced beta-oxidation of TG, leading to reduced TG levels. Concurrently, we observed alterations in serum T3 and T4 levels, as well as reductions in plasma tyrosine and phenylalanine concentrations, indicating a beneficial effect of this intervention on thyroid function. Transcriptomic analysis conducted under similar intervention conditions revealed that the regulation level of the thyroid hormone signaling pathway was significantly elevated compared to preintervention levels, with notable changes in gene expression primarily involving inhibition of oncogene expression and modulation of glycolytic activity, underscoring the body's adaptive mechanisms in response to metabolic changes. These findings highlight diet and exercise as pivotal modulators of thyroid health and metabolic efficiency. However, given the modest sample size, these results warrant further investigation through comprehensive prospective studies that include long-term follow-up assessments regarding the effects of these interventions across diverse populations.

## 5. Strengths and Limitations

Our study integrates two weight loss intervention strategies to systematically assess the combined effects of these interventions on obese individuals. Utilizing lipidomics and metabolomics techniques, we elucidated the potential impacts of weight loss on thyroid hormone levels. Furthermore, by analyzing public databases, we explored the possible mechanisms underlying this change, thereby providing a novel perspective and scientific foundation for obesity intervention. Despite achieving some significant results, there are still some limitations. First, our sample size was relatively small due to project constraints. Although the changes in body composition before and after the intervention were statistically significant, we cannot entirely dismiss the possibility of chance influencing these outcomes. Second, owing to limitations in sample collection methods, we only obtained blood samples from participants and did not encompass a broader range of tissue types. While we mitigated this bias by utilizing data from public databases under similar intervention conditions, the influence of different tissues on the results cannot be ignored.

## 6. Conclusion

Our research indicates that a weight loss approach combining dietary adjustments with exercise interventions has improved thyroid function in individuals with obesity. This improvement may be attributed to the enhanced consumption of tyrosine and phenylalanine, as well as an increased activity of the thyroid hormone signaling pathways within the body. These findings provide valuable insights into potential therapeutic strategies for addressing thyroid dysfunction associated with obesity.

## Figures and Tables

**Table 1:** Basic characteristics of volunteers and serological changes before and after intervention.

	Before		After		*p* value
	Mean	SD	Mean	SD	
WC (cm)	105.319	15.55	100.81	15.00	< 0.001
W (kg)	98.21	21.53	93.73	19.96	< 0.001
BMI (kg/m^2^)	34.03	6.38	32.34	5.96	< 0.001
VTA (cm^2^)	154.29	57.94	120.14	47.42	0.001
AST (U/L)	44.90	48.14	45.02	31.54	0.152
ALT (U/L)	48.77	50.30	50.68	46.47	0.808
FPG (mmol/L)	6.01	1.64	4.25	0.62	0.001
TG (mmol/L)	1.85	1.20	1.06	0.37	0.016
TC (mmol/L)	4.82	0.54	4.23	0.84	0.001
LDL-C (mmol/L)	2.71	0.39	2.46	0.67	0.072
FT3 (pmol/L)	5.59	1.69	4.20	0.74	0.004
FT4 (pmol/L)	16.21	2.07	17.58	2.07	0.005
FT3/FT4	0.34	0.08	0.24	0.05	< 0.001
TSH (uIU/mL)	2.66	1.40	2.25	1.13	0.187

*Note:* W: body weight, AST: aspartate aminotransferase, ALT: alanine aminotransferase, TG: triglyceride, FT3: triiodothyronine, FT4: free thyroxine, FT3/FT4: triiodothyronine/free thyroxine.

Abbreviations: BMI, body mass index; FPG, fasting plasma glucose; LDL-C, low density lipoprotein cholesterol; TC, total cholesterol; TSH, thyroid stimulating hormone; VTA, visceral fat area; WC, waist circumference.

## Data Availability

All datasets generated and analyzed during the current study are not publicly available but are available from the corresponding authors upon reasonable request.

## Supporting Information

Supporting Figure 1. PCA of the lipidomic data. Supporting Figure 2. Mean changes after intervention for different classes of phospholipids. Supporting Figure 3. Changes in lysophospholipids before and after intervention. Supporting Figure 4. Changes in sphingolipid before and after intervention. Supporting Figure 5. Results of PCA and OPLS-DA. Supporting Figure 6. Box plots depicting MED13 changes in standard care. Supporting Figure 7. Box plots depicting HRAS changes in standard care. Supporting Figure 8. Box plots depicting PFKFB2 changes in standard care.
