# Supplementary material for: Serum Lipidome and Metabolome Alterations in Obese Patients Undergoing Targeted Diet and Exercise Interventions: A Marker of Thyroid Function Recovery?
Source: Int J Endocrinol. 2025 Sep 18;2025:6065721. doi: 10.1155/ije/6065721 (PMC12463530; doi:10.1155/ije/6065721)

**Legends of figures**

**Supplemental Figure 1. PCA of the lipidomic data**

**Supplemental Figure 2. Mean changes after intervention for different classes of phospholipids**

Abbreviation: PC: phosphatidylcholine, PE: phosphatidylethanolamine, PI: phosphatidylinositol, PG: phosphatidylglycerol, PS: phosphatidylserine, *P < 0.05.

**Supplemental Figure 3. Changes in lysophospholipids before and after intervention**

Abbreviation: LPE: lysophosphatidylethanolamine, LPI: lyysophosphatidylinositol, LPS: lysophosphatidylserine, LysoPC: lysophosphatidylcholine, *P < 0.05.

**Supplemental Figure 4. Changes in Sphingolipid before and after intervention**

Abbreviation: SM: sphingolipid, Cer: ceramide, *P < 0.05.

**Supplemental Figure 5. Results of PCA and OPLS-DA**

1. Significance diagnosis: Scatter plot of the R2Y and Q2Y values of the real and simulated models randomly arranged, when the model R2Y and Q2Y(scatter) are greater than the true value (horizontal line), indicating overfitting; (B) Inertia bar chart: The adequacy of the orthogonal component was assessed by showing the cumulative explanatory rate; (C) Outliers: The distances of each sample in the projection plane as well as in the orthogonal projection plane are shown. Colors represent intervention groups; (D) X-score plot: The coordinates of each sample in the OPLS-DA axis, and the colors represent the groups; (E) Plot of principal component scores.

**Supplemental Figure 6. Box Plots Depicting MED13 Changes in Standard Care**

Abbreviation: SC: standard care.

**Supplemental Figure 7. Box Plots Depicting HRAS Changes in Standard Care**

Abbreviation: SC: standard care.

**Supplemental Figure 8. Box Plots Depicting PFKFB2 Changes in Standard Care**

Abbreviation: SC: standard care.

**Supplemental Figure 1. PCA of the lipidomic data**


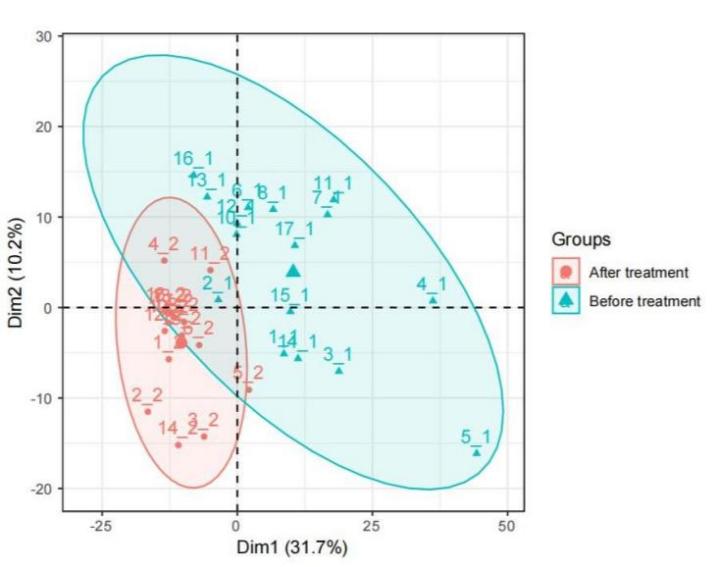


**Supplemental Figure 2. Mean changes after intervention for different classes of phospholipids**


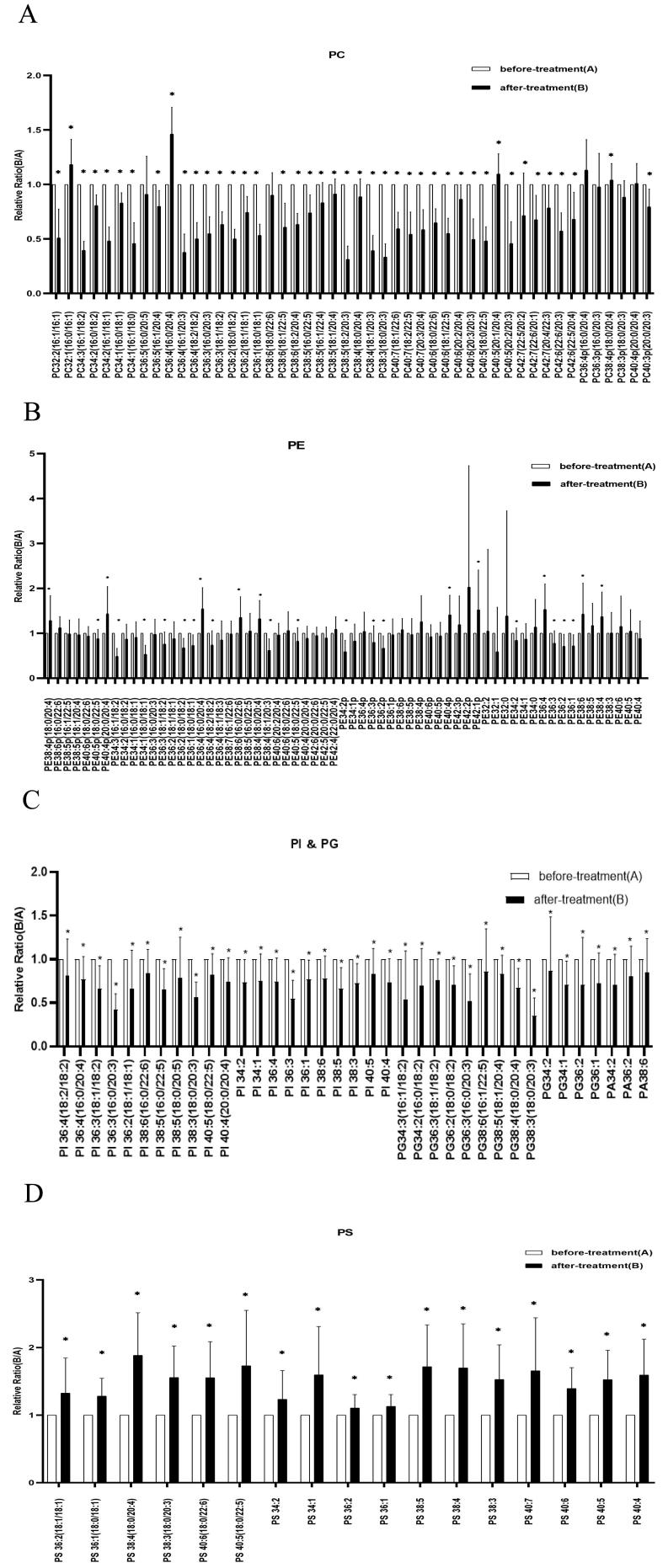


**Supplemental Figure 3. Changes in lysophospholipids before and after intervention**


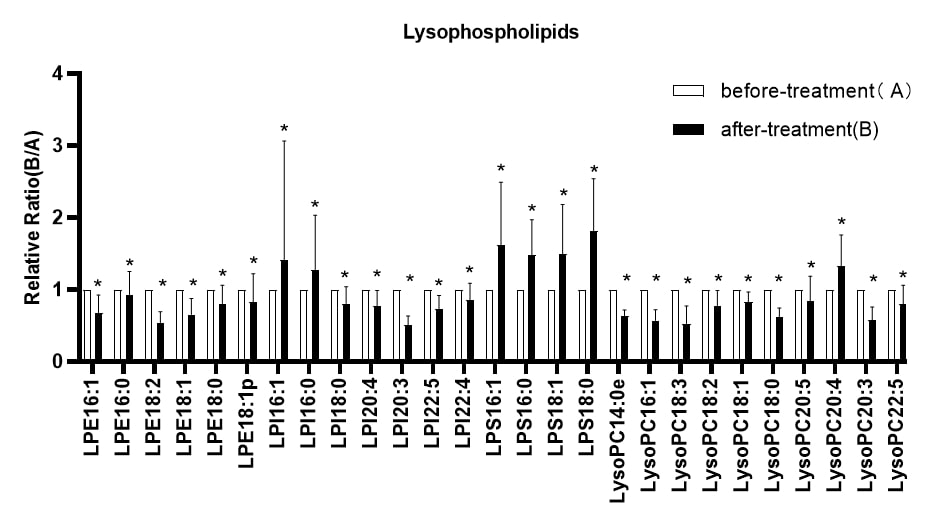


**Supplemental Figure 4. Changes in Sphingolipid before and after intervention**


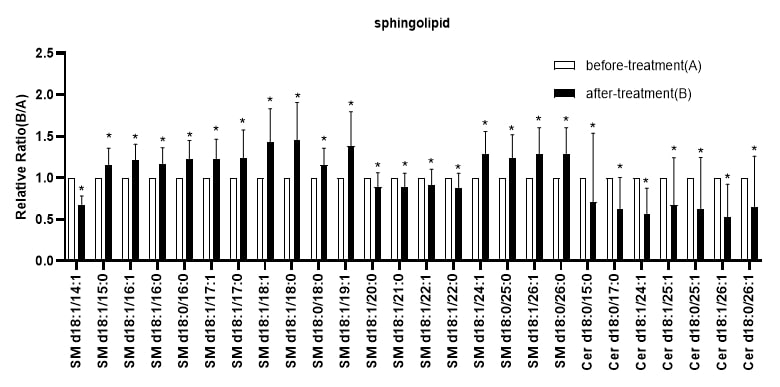


**Supplemental Figure 5. Results of PCA and OPLS-DA**


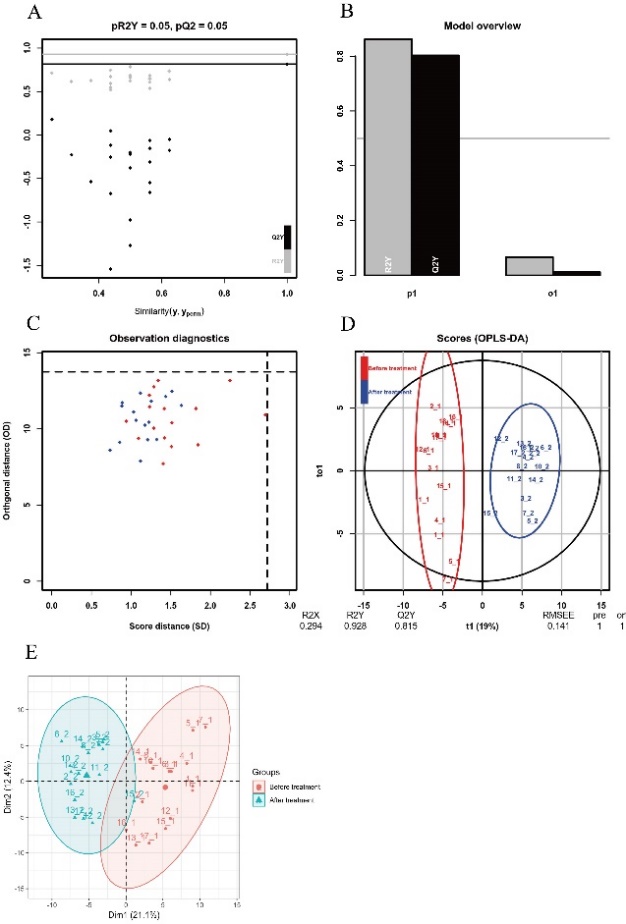


**Supplemental Figure 6. Box Plots Depicting MED13 Changes in Standard Care**


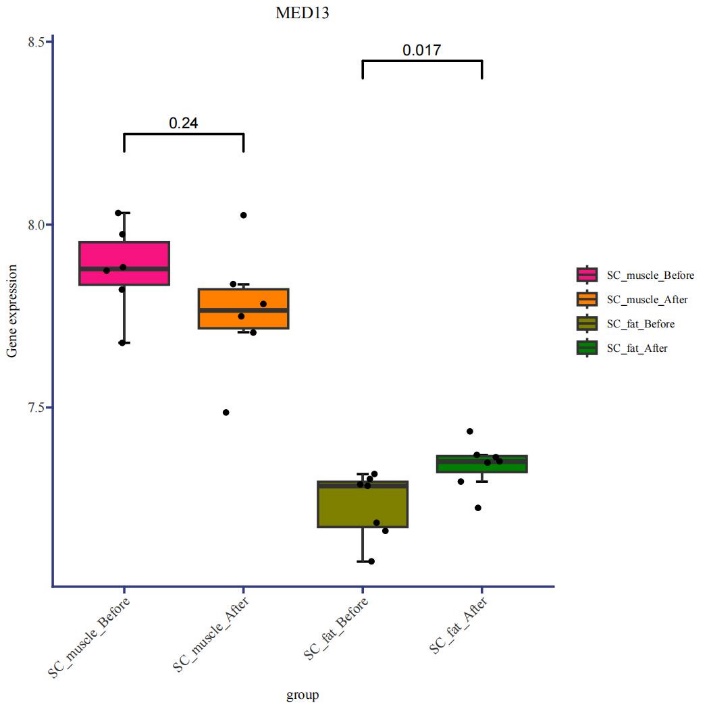


**Supplemental Figure 7. Box Plots Depicting HRAS Changes in Standard Care**


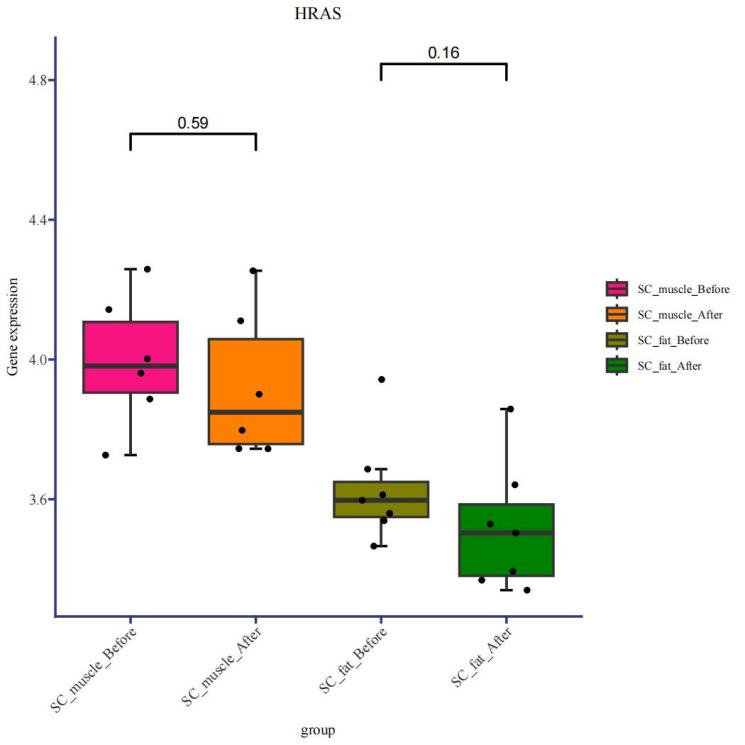


**Supplemental Figure 8. Box Plots Depicting PFKFB2 Changes in Standard Care**


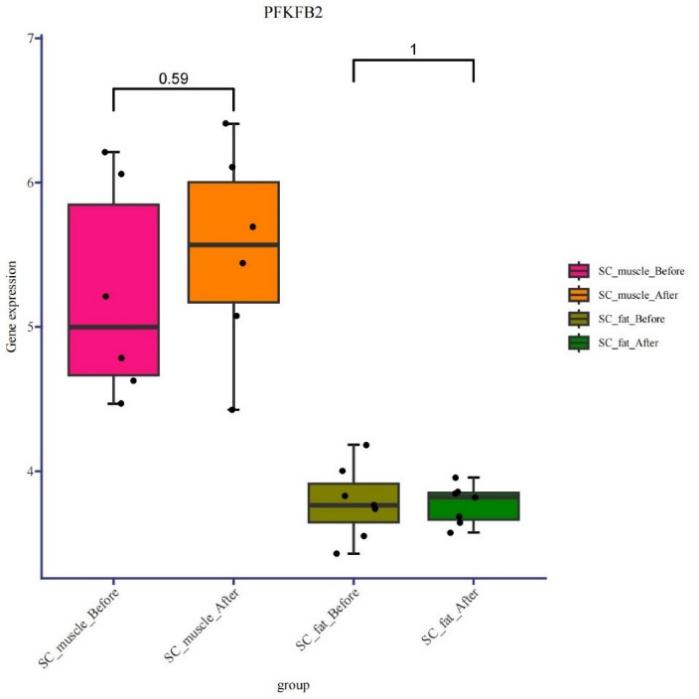

Supplement: Supporting Information — Additional supporting information can be found online in the Supporting Information section. [file 6065721.f1.docx]
